# Renal-Protective Roles of Lipoic Acid in Kidney Disease

**DOI:** 10.3390/nu15071732

**Published:** 2023-04-01

**Authors:** Sulin F. Kamt, Jiankang Liu, Liang-Jun Yan

**Affiliations:** 1 Department of Pharmaceutical Sciences, College of Pharmacy, University of North Texas Health Science Center, Fort Worth, TX 76107, USA; sulinkamt@my.unthsc.edu; 2 School of Health and Life Sciences, University of Health and Rehabilitation Sciences, Qingdao 266071, China; jkliu@uor.edu.cn; 3 Center for Mitochondrial Biology and Medicine, The Key Laboratory of Biomedical Information Engineering of Ministry of Education, School of Life Science and Technology, Xi’an Jiaotong University, Xi’an 710049, China

**Keywords:** lipoic acid, acute kidney injury, chronic kidney disease, diabetic kidney disease, diabetic nephropathy, nephroprotection

## Abstract

The kidney is a crucial organ that eliminates metabolic waste and reabsorbs nutritious elements. It also participates in the regulation of blood pressure, maintenance of electrolyte balance and blood pH homeostasis, as well as erythropoiesis and vitamin D maturation. Due to such a heavy workload, the kidney is an energy-demanding organ and is constantly exposed to endogenous and exogenous insults, leading to the development of either acute kidney injury (AKI) or chronic kidney disease (CKD). Nevertheless, there are no therapeutic managements to treat AKI or CKD effectively. Therefore, novel therapeutic approaches for fighting kidney injury are urgently needed. This review article discusses the role of α-lipoic acid (ALA) in preventing and treating kidney diseases. We focus on various animal models of kidney injury by which the underlying renoprotective mechanisms of ALA have been unraveled. The animal models covered include diabetic nephropathy, sepsis-induced kidney injury, renal ischemic injury, unilateral ureteral obstruction, and kidney injuries induced by folic acid and metals such as cisplatin, cadmium, and iron. We highlight the common mechanisms of ALA’s renal protective actions that include decreasing oxidative damage, increasing antioxidant capacities, counteracting inflammation, mitigating renal fibrosis, and attenuating nephron cell death. It is by these mechanisms that ALA achieves its biological function of alleviating kidney injury and improving kidney function. Nevertheless, we also point out that more comprehensive, preclinical, and clinical studies will be needed to make ALA a better therapeutic agent for targeting kidney disorders.

## 1. Introduction

The kidney is a vital organ, participating in maintenance of electrolyte balance, blood pH stability, removal of metabolic waste, and reabsorption of nutrients and minerals [1,2]. The kidney is also involved in erythropoiesis, vitamin D maturation, and blood pressure regulation [3]. Under pathophysiological conditions such as fasting, long-term starvation, and insulin resistance, the kidney can also regenerate glucose via gluconeogenesis using precursor molecules such as glycerol, alanine, pyruvate, and lactate [4]. As such, the kidney always has a heavy workload and is thus exposed to numerous risk factors that can cause kidney disease or injuries. There are two types of kidney diseases: chronic kidney disease (CKD) [5] and acute kidney injury (AKI) [6]. CKD occurs when there’s a gradual decline in renal function for more than 3 months due to damage to glomerular filtration and tubular injuries [7]. It has been projected that by 2040, CKD will become the fifth leading cause of death worldwide [8]. On the other hand, AKI occurs when there’s a rapid decline in renal function for less than 3 months, and this is indicated by acidosis, fluid overload, and abnormalities with electrolytes and hematology changes [7]. It is also known that individuals with CKD can have an increased risk of AKI [8]. Currently, there are no pharmaceutical products to cure AKI or CKD. While renal dialysis is often used to prevent further damage to the kidneys and maintain their function, renal replacement may be the last resort for patient survival. Therefore, if uncontrolled, AKI and CKD can lead to kidney failure, which significantly increases morbidity and mortality [7,9]. Hence, there is an unmet need in fighting kidney disease. 

**Figure 1:**
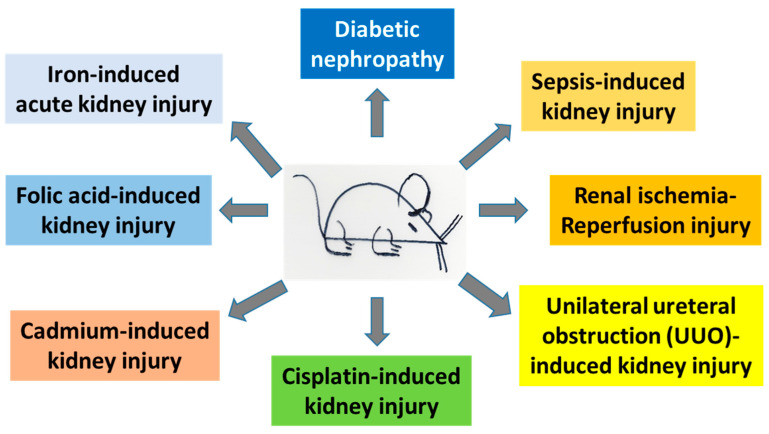
Animal models of kidney injury discussed in this article. These models include both acute kidney injury and chronic kidney disease.

To fight kidney diseases, numerous therapeutic approaches have been explored [10,11,12,13]. These include exogenous and endogenous compounds, dietary manipulations, modulation of metabolic pathways, stem cell approaches, and cell signaling processes [10,11,14,15,16,17,18,19,20,21]. In this review article, we will focus on the role of lipoic acid in preventing and ameliorating kidney injuries. In particular, we will focus on studies using animal models for exploring the protective effects of lipoic acid on kidney injuries. These animal models of kidney injury include diabetic kidney disease or diabetic nephropathy, renal ischemia-reperfusion injury, sepsis-induced kidney injury, unilateral ureteral obstruction (UUO)-induced kidney injury, cisplatin-induced kidney injury, cadmium-induced kidney injury, folic acid-induced kidney injury, and iron-induced acute kidney injury (Figure 1).

## 2. Alpha-Lipoic Acid

**Figure 2:**
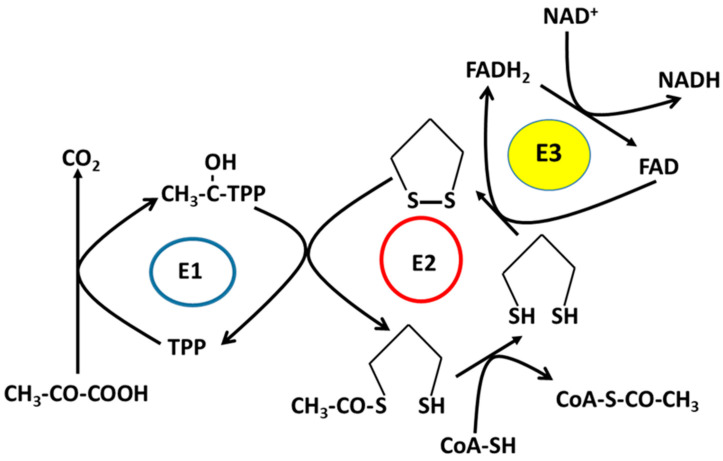
Lipoic acid is a cofactor of mitochondrial 2-ketoacid dehydrogenase complex, including pyruvate dehydrogenase complex, α-ketoglutarate dehydrogenase complex, and branched-chain amino acid dehydrogenase complex. The E1 subunit is 2-ketoacid decarboxylase using TPP as a cofactor; the E2 subunit is a dihydrolipoamide acyltransferase using lipoic acid as a cofactor; the E3 subunit is a dihydrolipoamide dehydrogenase, which uses NAD^+^ as an electron acceptor for the oxidation of the lipoyl group linked to the E2 subunit. E3 catalyzes the formation of the oxidized form of lipoic acid and generates NADH in the meantime [32,33,34].

**Figure 3:**
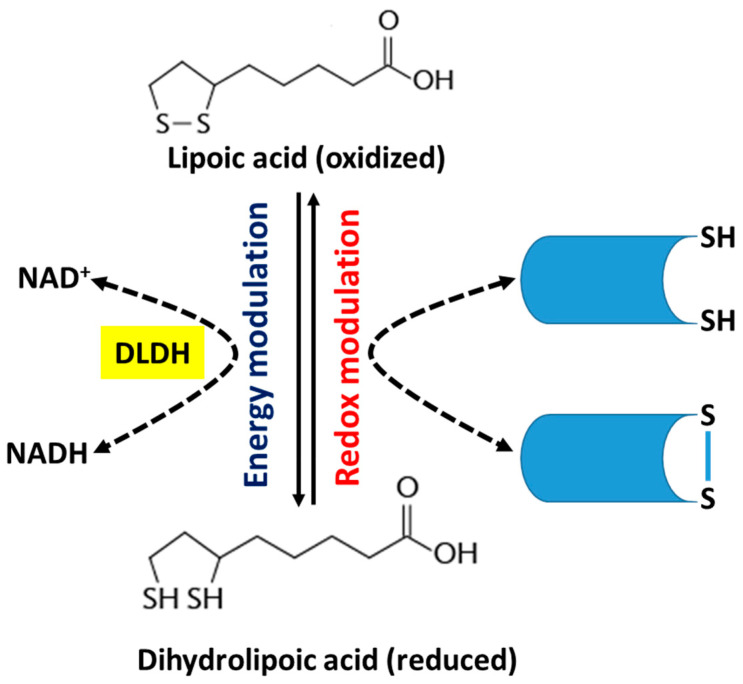
Lipoic acid is involved in thiol-disulfide exchanges that modulate cell’s redox and energy status.

**Figure 4:**
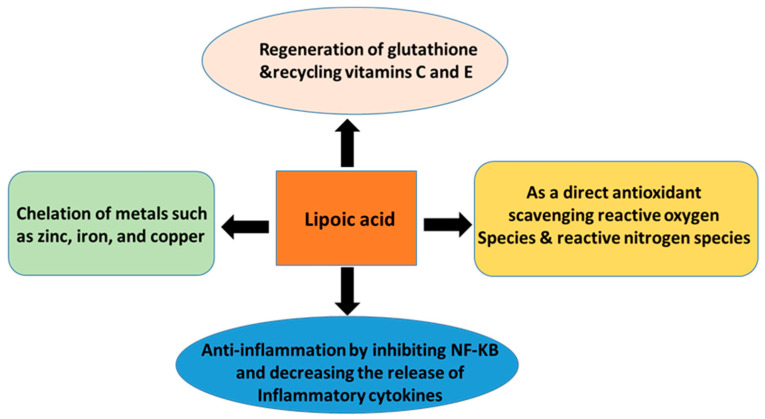
Biological actions of lipoic acid and its potential mechanisms, which include regeneration of glutathione, vitamins C and E, scavenging ROS, chelating metal ions, and anti-inflammatory power.

ALA is a naturally occurring dithiol compound [22]. It is a cofactor for α-ketoglutarate dehydrogenase, branched-chain amino acid dehydrogenase, and pyruvate dehydrogenase [23,24,25] (Figure 2). Therefore, ALA is an energy modulator [26,27] (Figure 3). Moreover, due to its capability of exchanging thiol groups with other thiol-containing molecules, such as glutathione and protein’s cysteine residues, ALA is also known as a redox modulator [26,27,28] (Figure 3). ALA is often referred to as a universal antioxidant because it can act as an antioxidant in both lipophilic and hydrophilic settings to reduce byproducts of oxidative metabolism, such as reactive oxygen species (ROS) and reactive nitrogen species (RNS) (Figure 4) [29]. ALA can also chelate metals such as zinc, iron, and copper and regenerate endogenous antioxidants—such as glutathione—and exogenous vitamin antioxidants—such as vitamins C and E—with minimal side effects [30] (Figure 4). More importantly, ALA can also inhibit inflammation by targeting NF-KB and decreasing the release of inflammatory cytokines (Figure 4). Therefore, it has been demonstrated that by scavenging oxygen free radicals, ALA could increase glomerular function and decrease renal inflammation [31]. Studies have also shown that treatment with ALA can decrease acute kidney injury by lowering serum blood urea nitrogen, creatinine levels, tumor necrosis factor-alpha (TNF-α), interleukin-6 (IL-6), and interleukin-1 beta (IL-1β), thereby decreasing endothelin-1 vasoconstriction, neutrophil diffusion, and inflammation in the kidneys.

## 3. Protective Roles of α-Lipoic Acid (ALA) in Kidney Injury

Kidney disorders can be induced by a number of insults, such as diabetes, ischemic reperfusion, drug toxicity, contrast media, and medications [7]. If not controlled, AKI can lead to renal failure with a 20% mortality rate [25]. AKI is characterized by an increase in serum creatinine, oliguria, and the presence of kidney damage markers such as albuminuria, electrolyte abnormalities due to tubular disorders, or structural damage seen in imaging or histology [9]. ALA prophylaxis has been shown to decrease renal tubular injury scores, urinary damage markers, serum creatinine structural damage, and increase glomerular filtration [35]. In the following sections, we will discuss the nephroprotective effects and the underlying protective mechanisms of ALA in a variety of animal models of kidney injury, as indicated in Figure 1.

### 3.1. Diabetic Nephropathy

**Figure 5:**
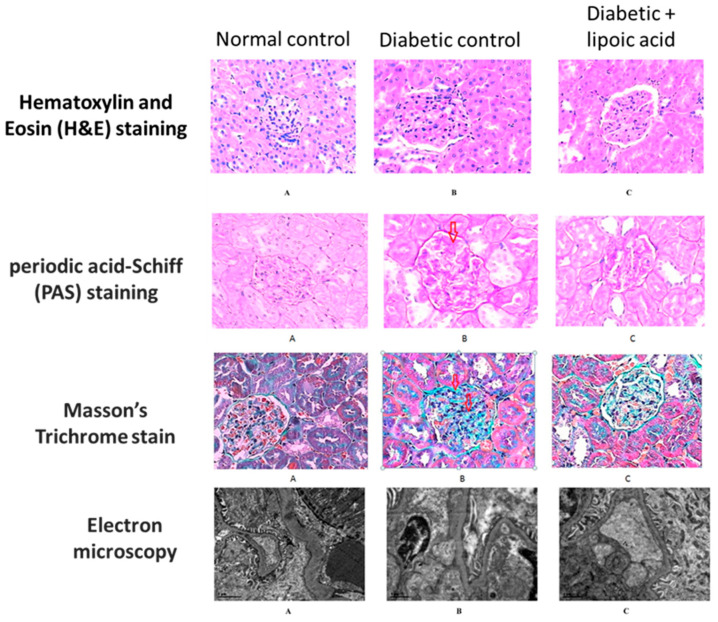
Protective effects of lipoic acid on diabetic kidney disease. Histological staining of the kidney tissues derived from control, diabetic, and diabetic + lipoic acid treatment. This figure was reproduced from reference [55]. For all the microscopic images, the amplification magnitude is ×800. (**A**) normal control group; (**B**) diabetic control group; (**C**) diabetic + ALA group. Glomerular hypertrophy, mesangial region expansion, proliferation of mesangial cells, and inflammatory cell filtration could also be observed in H&E staining. Disruption of glomerular basement membrane and mesangial region expansion could also be observed in PAS staining. Additionally, collagen fiber staining shows an increased intensity in the diabetic control group but exhibits a significant decrease in ALA-treated animals. For PAS staining, the red arrow indicates thickening and deformation in the glomerular basement membrane in the diabetic kidney. For Masson’s stained sections, the red arrows indicate the increased intensity of collagen fiber stain in the diabetic kidney.

Diabetic nephropathy (DN), also known as diabetic kidney disease (DKD) [36,37,38], is a major cause of CKD and end-stage kidney failure in diabetic patients [39,40,41]. It has been well-established that mitochondrial dysfunction contributes to DKD, and the mitochondrion is a target for fighting DKD [42,43,44]. DN can be considered a microvascular complication from type 1 or type 2 diabetes mellitus [7,41]. This diabetic kidney disease is characterized by a decrease in glomerular filtration, proteinuria, and renal fibrosis [7]. Hyperglycemia increases oxidative stress, leading to the early overproduction of reactive oxygen species (ROS) [45] and dysregulation of glutathione metabolic pathways [7]. Malondialdehyde (MDA) is the end product of lipid peroxidation and has been commonly used as a good marker of free radicals and oxidative stress [46,47,48,49]. Studies have shown that pretreatment with ALA decreased MDA content and ameliorated renal oxidative stress [32,50]. Lipoamide, a derivative of ALA, has been demonstrated to inhibit kidney fibrosis in diabetes by enhancing mitochondrial function and regulating the expression of activation of transcriptional factor retinal X receptor alpha [51]. In a rat model of diabetes induced by nicotinamide together with streptozotocin, a less time-consuming approach in creating rodent models of diabetes [52], Dugbartey et al. have also demonstrated that ALA’s renal protective mechanism involves activation of the renal cystathionine γ-lyase/hydrogen sulfide system [53]. It has also been demonstrated that ALA has a synergistic effect on attenuating serum levels of inflammatory cytokines and improving kidney function in diabetic animals when combined with the angiotensin II receptor inhibitor valsartan [54]. Figure 5 shows compelling evidence of histological staining that ALA exhibits strong protective effects on diabetic kidneys in a type 2 diabetes animal model [55]. Another potential mechanism of ALA’s nephroprotection in diabetes is its ability to activate the Nrf2 signaling pathway, leading to upregulation of the second-phase cytoprotective proteins such as heme oxygenase-1 (HO-1) and NAD(P)H quinone dehydrogenase 1 (NQO1) [56,57,58]. It should be pointed out that while ALA is nephroprotective in diabetic kidney disease, it may impose pro-oxidant or toxic effects and could fail to serve as an Nrf2 inducer under certain pathophysiological conditions [59,60,61]. Additionally, while ALA has been thought to activate insulin signaling pathways to combat diabetes [28] and has been shown to prevent high fructose-induced cardiometabolic disorders and renal dysfunction [62], it has also been reported that ALA could only attenuate proteinuria and oxidative stress without slowing progression of diabetic renal failure [63].

### 3.2. Sepsis-Induced Kidney Injury

Sepsis is potentially a life-threatening pathological condition due to an overdriving inflammatory response to bacterial infection [64,65,66]. Severe sepsis can cause multi-organ failures, with the kidney being the most-affected organ [67,68]. It has been estimated that nearly 50% of septic patients would develop acute kidney injury, and there are no effective treatments for septic AKI [69]. In this regard, numerous investigators have comprehensively evaluated ALA’s protective and therapeutic values in sepsis-induced AKI modeled by injection of lipopolysaccharides, though cacal puncture and ligation (CPL)-induced septic AKI have been occasionally used [22,70]. It has been demonstrated that ALA can protect against septic kidney injury by enhancing autophagy [71]. Moreover, ALA may also ameliorate sepsis-induced AKI by counteracting inflammation via inhibition of the NF-KB signaling pathway [72], attenuating mitochondrial oxidative stress, and preserving the type 3 Na^+^/H^+^ exchanger and aquaporin 2 expression in the kidney [73]. ALA can also inhibit the release of tumor necrosis factor-α, interleukin (IL)-6, and IL-1β into the serum and suppress the expression of inducible nitric oxide synthase [22] in septic AKI. Therefore, ALA could be a promising natural product for the treatment of septic AKI.

### 3.3. Renal Ischemic Reperfusion

Ischemia occurs when there is a decrease in blood perfusion and the blood flow to organs is reduced [74]; this is due to many causes, such as thrombi, trauma, and atherosclerosis [75]. To prevent tissue damage and necrosis, ischemia is resolved by reperfusion [75]. Although ischemic reperfusion is essential to prevent tissue necrosis, it can also cause inflammation and an increase in reactive oxygen species and reactive nitrogen species [75,76,77]. Studies have shown that pretreatment of ALA can ameliorate damage to the kidneys, retina, nervous system, liver, intestines, and more [75]. The underlying protective mechanisms of ALA in renal ischemia reperfusion injury may be multifactorial, including counteracting oxidative damage [32,75] and downregulation of channels, enzymes, and transporters, such as aquaporins and sodium transporters, as well as sodium-potassium ATPase and nitric oxide synthase isoforms [78]. ALA may also protect renal ischemia reperfusion injury by mitigating neutrophil infiltration and inhibiting the release of inflammatory mediators [79]. Studies have also demonstrated that renal cortical structure damage induced by limb ischemia-reperfusion injury can be ameliorated by ALA [80] and ALA, and when combined with the xanthine oxidase inhibitor febuxostat, shows superior protective effects on renal ischemia reperfusion injury [81]. ALA may also prevent renal dysfunction and kidney injury by suppressing the overexpression of endothelin-1 in renal ischemia reperfusion injury [82].

### 3.4. Unilateral Ureteral Obstruction (UUO)-Induced Kidney Injury

The unilateral ureteral obstruction (UUO) animal model of kidney injury has been widely used to investigate the mechanisms of kidney injury and the therapeutic values of numerous agents [83,84,85,86,87]. This model has certain advantages in that it is a nonuremic normotensive disorder in the absence of any apparent inflammatory or toxic insult to the kidneys [88]. Moreover, the UUO model of kidney injury is also a good model for studying the pathophysiology of renal fibrosis [89,90,91,92,93]. Therefore, the UUO model of kidney injury may closely mimic the underlying pathophysiology of human obstructive kidney injury [94,95]. ALA has been shown to be renoprotective against UUO-induced kidney injury [88]. Wongmekiat et al. have found that when ALA (60/mg/kg body weight) was given to rats via i.p. injection two days before UUO induction and continued for a week after UUO, UUO-induced renal dysfunction, oxidative stress, and production of nitric oxide and transforming factor-1 were greatly attenuated by ALA treatment [88]. Additionally, ALA has also been demonstrated to ameliorate the epithelial-mesenchymal transition in a mouse model of UUO renal injury [96]. These studies thus demonstrate that ALA is nephroprotective against UUO-induced kidney injury.

### 3.5. Cisplatin-Induced Nephrotoxicity

Cisplatin is a chemotherapy drug that treats cancer by entering the tumor cells, releasing chloride ions, and becoming hydrated to crosslink with DNA and form DNA adducts to inhibit tumor cell replication [97,98]. However, cisplatin medication has many side effects, such as ototoxicity, neurotoxicity, nephrotoxicity, nausea, and vomiting [35,97,98,99]. Nephrotoxicity occurs because cisplatin is taken up by the proximal tubular cells and can concentrate up to five times more than the cisplatin in the serum [35,97]. Cisplatin nephrotoxicity leads to decreased creatinine clearance, increased serum creatinine levels, increased urea levels, increased urine output, and decreased glomerular filtration rate [100,101]. Cisplatin also decreases the antioxidants glutathione S-transferase, glutathione peroxidase, and superoxide dismutase, causing an elevation in ROS and oxidative markers such as MDA derived from lipid peroxidation [35,100,102].

Proximal tubules in the kidneys contain large amounts of mitochondria [35]. Hydrolyzed cisplatin creates a metabolite with a positive charge, and this accumulates within mitochondria due to mitochondria’s negatively charged molecules, creating high levels of oxidative stress [35]. To manage this, mitochondria use endogenous antioxidants such as lipoic acid to reduce ROS [35]. Studies have shown that ALA protects renal cells against cisplatin’s toxicity [98], decreases structural proximal tubular damage, and increases glomerular filtration in kidneys [99,103]. ALA has also been shown to lower plasma creatinine levels and urine output, increase creatinine clearance and urine osmolality, and normalize sodium excretion in cisplatin kidney injury [101].

### 3.6. Folic Acid-Induced Nephrotoxicity

**Figure 6:**
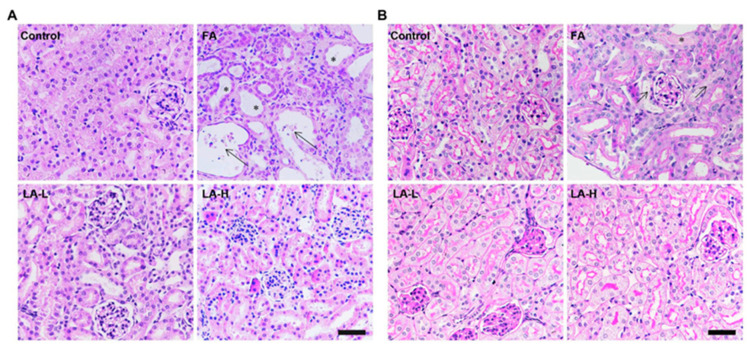
Protective effects of lipoic acid on folic acid-induced kidney injury. Histological staining of the kidney tissues derived from control, folic acid (FA), and folic acid + lipoic acid (LA). This figure was reproduced from reference [105]. (**A**) Representative images of hematoxylin & eosin (H&E) staining reflecting the histological changes in folic acid-induced acute kidney injury and low- and high-ALA-treated animal groups. (**B**) Renal damage assessed by Periodic acid/Schiff staining in folic acid-induced acute kidney injury and low- and high-ALA-treated animal groups.

High levels of folic acid can cause tubular damage by the detachment and dilation of tubular cells [104], leading to cell death [105]. Ferroptosis is a type of cell death caused by iron and lipid peroxidation that occurs during folic acid-induced AKI [106]. When large amounts of iron are present in the body, ROS are generated, causing lipid peroxidation, damaging the lipid membranes, and causing cell death [105]. It has been established that ALA’s antioxidant effects resulted in renoprotection against folic acid-induced kidney damage [105]. In the study by Li et al., there was no significant difference between low doses and high doses of ALA, indicating that the benefits of ALA are not dose-dependent [105]. Furthermore, ALA may work by blocking p53 from causing ferroptosis and by upregulating ferritin and ferroportin iron exporters, thus preventing folic acid-induced AKI [105]. Figure 6 shows histologically the visualization of ALA protection against renal injury induced by folic acid [105].

### 3.7. Cadmium-Induced Nephrotoxicity

Cadmium is a naturally occurring toxic heavy metal that causes nephrotoxicity [104,107,108,109]. Cadmium-induced nephrotoxicity increases MDA levels, causing damage to renal mitochondria and the renal cortex [110]. Cadmium has also been shown to decrease glutathione antioxidant (GSH), glutathione peroxidase, catalase, and superoxide dismutase (SOD) [110]. Treatment with ALA functioned as an antioxidant to decrease MDA and apoptosis and chelate cadmium to lower cadmium damage to the kidneys [111]. ALA also lowered oxidative stress, promoted glutathione-related endogenous enzymes, and prevented mitochondrial apoptosis in cadmium-induced kidney injury [110,111,112].

### 3.8. Iron-Induced Acute Kidney Injury

Iron has been thought to contribute to both AKI and CKD [113,114,115,116,117,118]. Indeed, it has been observed that renal tubules are exposed to elevated levels of iron in patients with kidney disease, which is likely due to increased filtration of iron and iron-containing proteins through the glomerular apparatus [114,117,119]. Iron can also aggravate diabetic kidney disease by increasing oxidative stress [120,121]. Therefore, iron-induced animal models of kidney injury have been a valuable tool in studying the mechanisms of iron-induced kidney injury and testing the therapeutic effects of natural products or pharmaceutical drugs [122,123,124,125]. In this regard, ALA has been shown to be nephroprotective in ion-induced kidney injury [126,127]. In an iron-overloading rat model of kidney injury, ALA was found to exhibit antioxidant effects by attenuating oxidative damage [128]. ALA was also found to inhibit p38 MAPK signaling and NADPH oxidase 4 expression in iron-induced kidney injury [127]. It should be noted that all of these protective effects of ALA in iron-induced kidney injury may be partly due to its iron-chelating property [128,129,130,131], which lowers the availability of free iron.

## 4. Miscellaneous

1. Aging is linked to functional decline in the kidneys [132,133]. It has been established that there are numerous changes in the molecular, structural, and morphological levels in the kidney [134,135]. In aging kidneys, there are increased levels of oxidative stress, as reflected by increased lipid peroxidation, mitochondrial dysfunction, and decreased levels of antioxidants, including superoxide dismutase, catalase, and glutathione peroxidase [136,137,138,139]. Not surprisingly, ALA could alleviate all of these deleterious changes in aged kidneys [136,137,138]. Additionally, ALA given as a dietary supplement has been shown to be able to reverse age-related decline in kidney function and serum total proteins [140]. These studies demonstrate the protective and preventative effects of ALA on kidney aging.

2. Sleep apnea is a disorder that causes intermittent hypoxia, which can further cause hypoxia-associated renal injury [141,142,143,144]. ALA has been found to be protective against renal injury induced by sleep apnea hypoxia [145]. In a study using a mouse model of sleep apnea, Abuyassin et al. have demonstrated that in the animals that underwent intermittent hypoxia and were treated with an ALA-enhanced diet, renal oxidative stress and inflammation were lower than those exposed to intermittent hypoxia only. Moreover, renal cell death and tubular injury were also deceased in the intermittent hypoxia + ALA group, and treatment with ALA mitigated intermittent hypoxia-induced glomerular hypertrophy and decreased albuminuria [145]. Therefore, ALA is nephroprotective in hypoxia-related kidney injury induced by sleep apnea.

3. Functional impairment of the kidneys is also a frequent disorder in the presence of high blood pressure, also known as hypertension. It has been thought that renal damage linked to hypertension is caused by oxidative stress [146,147,148]. In this regard, ALA has also been tested for its antioxidant role in hypertension-induced kidney injury. Martinelli et al. [149] have found that when spontaneous hypertensive rats with high blood pressure were treated by a racemic mixture of ALA, renal oxidative damage was attenuated, with a significant improvement in kidney function accompanied by ameliorated glomerular and tubular injury. This study also indicates that when exogenous ALA is administered, a racemic mixture of ALA is often used [149].

4. Chronic kidney disease (CKD) can be created by feeding animals with a high concentration of adenine [150,151,152]. In fact, adenine-induced CKD is a popular model for studying the pathophysiology of chronic kidney injury and the therapeutic effects of a variety of natural products or drugs [153,154,155]. Nonetheless, the nephroprotective role of ALA has not been comprehensively evaluated in this animal model, which should be investigated in the future. Additionally, the protective effects of ALA on each of the five stages of chronic kidney disease [156,157,158] should also be studied in this animal model of CKD.

5. IgA nephropathy (IgAN) is known to cause glomerulonephritis due to deposition of IgA 1 [159,160,161] and is a prevalent chronic kidney disease [162,163,164]. Its major feature is mesangial cells and mononuclear leukocyte infiltration in renal interstitial tissues and the glomerulus. It has been demonstrated that oxidative stress-induced protein oxidation and lipid peroxidation is one of the underlying pathogenic mechanisms [165,166,167,168,169]. While animal models of IgAN are available for studying the pathogenesis of IgAN and exploring therapeutic approaches [170,171], the potential effects of ALA in this kidney disorder have not been evaluated. Nonetheless, it is conceivable that ALA would exhibit nephroprotective effects in IgAN, given its powerful antioxidant capacity.

6. It is also worth noting that studies comparing the renoprotective effect of ALA with other agents have also been conducted in recent years. For example, ALA was compared with a traditional Chinese medicine Huangkui capsule in rats with diabetic nephropathy [172]. The authors found that ALA is equivalent to the Huangkui capsule in renoprotection against diabetic kidney injury, and both agents improve kidney function by attenuating oxidative stress and downregulating the activation of the p38MAPK and Akt pathways. ALA has also been compared with *N*-acetylcysteine (NAC) in one study, whereby the authors found that NAC is better than ALA in protecting oxidative kidney injury induced by the chemotherapeutic drug ifosfamide, which is highly toxic to the kidney [173,174,175,176]. However, the authors used an NAC concentration (200 mg/kg) that was twice that of ALA (100 mg/kg) [176]. Hence, the conclusion that NAC is more renoprotective than ALA in this animal model of kidney injury may not be definitive. It should also be noted that a recent report indicates that ALA can also minimize renal toxicity induced by gold nanoparticles, which are often used as drug carriers [177]. In fact, poly(lipoic acid) nanoparticles themselves can also be used as a therapeutic tool for delivering active compounds [178].

## 5. Summary

**Figure 7:**
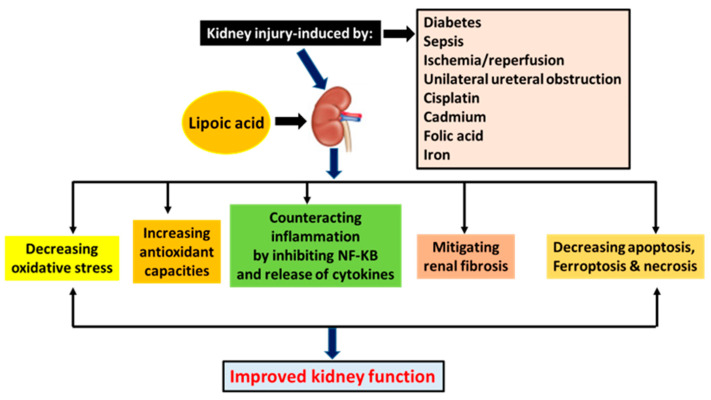
Mechanisms underlying lipoic acid’s role in various animal models of kidney injury discussed in the text. Lipoic acid alleviates kidney injury and improves kidney function by exerting various biological actions, as depicted in this figure.

In this article, we have reviewed the protective mechanisms of ALA in various animal models of kidney injury. These models cover both AKI and CKD, which include DKD, ischemia-reperfusion-induced kidney injury, sepsis-induced kidney injury, and kidney disorders induced by UUO, cisplatin, cadmium, folic acid, and iron. Common underlying mechanisms of ALA’s renoprotection are summarized in Figure 7.

As further summarized in Table 1, these mechanisms include decreasing oxidative stress, increasing endogenous antioxidant defense capacities, counteracting inflammation by inhibiting NF-kB and release of inflammatory cytokines, mitigating renal fibrosis, and decreasing cell death, such as apoptosis, ferroptosis, and necrosis. The eventual outcome of ALA treatment, regardless of the kidney injury models and the protective mechanisms unraveled, is the improvement of kidney function. Therefore, ALA is a promising agent targeting kidney disorders.

## 6. Future Perspectives

Most studies discussed in this review article utilized systemic administration of ALA in their studies of the protective effects of ALA on the respective animal models of kidney injury. Systemic administration of ALA will certainly result in distribution of ALA to the organs or tissues that may not need ALA, whereby excess ALA may pose deleterious effects [59]. Therefore, future studies will need to focus on developing approaches by which ALA will only be delivered to the kidneys. Such studies of target delivery of ALA to the kidney will certainly provide more insights into the protective mechanisms of ALA in different kidney injury models, elucidating both redox- and energy-modulatory properties of ALA. In this regard, nanoparticle delivery or nanomedicines of ALA targeting the kidney could be a promising approach [179,180,181,182,183,184]. Additionally, the combination of ALA with other natural products in treating kidney injuries and preventing the AKI to CKD transition will be interesting to investigate. Moreover, comprehensive preclinical and human studies are needed to evaluate the efficacy of ALA in the settings of AKI and CKD, as well as the AKI to CKD transition.

## Figures and Tables

**Table 1:** Mechanisms of ALA’s renal protection in various animal models of kidney injury discussed in this review.

Model of Kidney Injury	Mechanism	Reference
Diabetic kidney injury	Activating Nrf2, decreasing oxidative stress, enhancing mitochondrial function, and inhibiting fibrosis	[7,29,50,51]
Sepsis-induced kidney injury	Enhancing autophagy, inhibiting NF-KB, attenuating mitochondrial oxidative stress, inhibiting inflammatory cytokine release	[22,71,72,73]
Ischemia/reperfusion injury	Counteracting oxidative stress, downregulating Na-K-ATPase and NOS, mitigating neutrophil infiltration, inhibiting inflammation, and suppressing endothelin-1 upregulation	[75,78,79,80,81,82]
UUO-induced kidney injury	Attenuating oxidative stress, decreasing nitric oxide production, decreasing transforming factor-1 expression, and ameliorating mesenchymal transition	[88,96]
Cisplatin-induced kidney injury	Increasing glomerular filtration, lowering plasma creatinine levels, increasing creatinine clearance, and attenuating oxidative damage	[98,99,101,103]
FA-induced kidney injury	Blocking p53 from causing ferroptosis	[105]
Cadmium-induced kidney injury	Chelating cadmium, decreasing oxidative stress, elevating glutathione content, and decreasing apoptosis	[108,110,111,112]
Iron-induced kidney injury	Attenuating oxidative damage, inhibiting p38 MAPK, inhibiting NADPH oxidase4, and chelating iron	[126,127,128,129]

## Data Availability

Not applicable.
